# Hsp90 Selectively Modulates Phenotype in Vertebrate Development

**DOI:** 10.1371/journal.pgen.0030043

**Published:** 2007-03-30

**Authors:** Patricia L Yeyati, Ruth M Bancewicz, John Maule, Veronica van Heyningen

**Affiliations:** Medical Research Council Human Genetics Unit, Western General Hospital, Edinburgh, United Kingdom; The Jackson Laboratory, United States of America

## Abstract

Compromised heat shock protein 90 (Hsp90) function reveals cryptic phenotypes in flies and plants. These observations were interpreted to suggest that this molecular stress-response chaperone has a capacity to buffer underlying genetic variation. Conversely, the protective role of Hsp90 could account for the variable penetrance or severity of some heritable developmental malformations in vertebrates. Using zebrafish as a model, we defined Hsp90 inhibitor levels that did not induce a heat shock response or perturb phenotype in wild-type strains. Under these conditions the severity of the recessive eye phenotype in *sunrise*, caused by a *pax6b* mutation, was increased, while in *dreumes,* caused by a *sufu* mutation, it was decreased. In another strain, a previously unobserved spectrum of severe structural eye malformations, reminiscent of anophthalmia, microphthalmia, and nanophthalmia complex in humans, was uncovered by this limited inhibition of Hsp90 function. Inbreeding of offspring from selected unaffected carrier parents led to significantly elevated malformation frequencies and revealed the oligogenic nature of this phenotype. Unlike in *Drosophila,* Hsp90 inhibition can decrease developmental stability in zebrafish, as indicated by increased asymmetric presentation of anophthalmia, microphthalmia, and nanophthalmia and *sunrise* phenotypes. Analysis of the *sunrise pax6b* mutation suggests a molecular mechanism for the buffering of mutations by Hsp90. The zebrafish studies imply that mild perturbation of Hsp90 function at critical developmental stages may underpin the variable penetrance and expressivity of many developmental anomalies where the interaction between genotype and environment plays a major role.

## Author Summary

Genetic variation is not always expressed as a single consistent phenotype even in familial diseases. Unilateral malformations in paired organs, such as the failure of an eye to develop on one side only, also remind us that gene function is often modified by environmental factors. Following observations by others in fruit flies, we explored the underlying mechanisms for such phenotypic fluctuation, using zebrafish as a vertebrate model. Earlier work suggested involvement of chaperone proteins like Hsp90, which assist with normal protein folding during development and also work overtime to keep proteins functional in response to environmental stress. Using specific drugs at defined times in early development for the limited reduction of Hsp90 activity, we showed that different cryptic genetic variants could be revealed consistently in genetically distinct fish strains. Once uncovered, the frequency of these variants was increased by inbreeding, confirming the role of underlying genetic factors. Similarly, we could modify the phenotypic severity of some—but not all—known gene variants, worsening some and improving others. It emerged that the most susceptible variants were those carrying amino acid alterations, in which assisted protein folding may either restore near normal function or facilitate malfunction, thus worsening phenotype. This insight may allow us to prevent recurrent malformations by minimizing or perhaps even counteracting the effects of exposure to environmental stress during development.

## Introduction

Human malformations frequently show no clear Mendelian inheritance pattern, even when familial recurrence suggests a strong underlying genetic component. Such phenotypic variability is generally defined as incomplete penetrance or variable expressivity [1,2], and it may be influenced by genetic background as well as by environmental factors. Predicting phenotypic outcomes for such cases is often an impossible challenge in clinical genetics. As a corollary, it has become clear that robustness of the wild-type (WT) phenotype to extensive genetic and environmental variation may be ascribed to the complexity, and hence strong buffering capacity of gene networks and cellular surveillance mechanisms [3,4]. These homeostatic systems are of major clinical relevance as potential prophylactic and therapeutic targets. Understanding the molecular events that can alter the balance between cryptic and overt phenotypes is therefore an important endeavour.

Based on observations in *Drosophila* [5], we set out to assess the role of stress-response pathways, particularly of heat shock protein 90 (Hsp90) function, in phenotype modification, using zebrafish as a vertebrate model. Hsp90 proteins are environmentally responsive chaperones, encoded at multiple loci in vertebrates. Under normal conditions they assist the maturation and folding of newly synthesised proteins and escort metastable regulatory molecules such as kinases and steroid hormone receptors [6]. In response to mutation or environmental stress, additional Hsp90 capacity is required to maintain newly destabilized proteins in a folding competent state. An unexpected consequence of this dual role is that Hsp90 can become functionally limiting, so that overt phenotypes may be uncovered from previously cryptic genetic variants, in spite of Hsp90 being further induced under stress [7]. Two recent studies showed that Hsp90 perturbation affects nearly all body parts in *Drosophila* and numerous morphological phenotypes in *Arabidopsis* [5,8]. Most changes were recurrent in a strain-specific manner and enriched by subsequent inbreeding, suggesting that they arose from previously silent but preexisting genetic variants [5]. Recently, it was proposed that Hsp90 can affect the epigenetic state at specific loci, leading to phenotype modulation [9]. Interfering with Hsp90 buffering therefore has phenotypic consequences, which are dependent on underlying genetic and epigenetic components, although it is not clear whether some preexisting genetic change is required for epigenetic inheritance of the trait [7].

The Hsp90 loci identified in humans have distinct subcellular localisation: HSP90 alpha and beta, encoded by HSP90AA1 and HSP90AB1, are expressed in the cytoplasm; while GRP94, a glucose responsive form encoded by HSP90B1 resides in the endoplasmic reticulum; and there is a mitochondrial form, TRAP1 (TNF receptor-associated protein 1). Genetic studies in mice suggest that despite the close similarity between Hsp90aa1 and Hsp90ab1, these paralogues are not functionally redundant [10]. The cytosolic Hsp90 proteins are thought to work as homodimers, but in association with several cochaperones [11]. The homodimer binds and releases its “client” proteins, aided by cochaperones, in an ATP-driven cycle [12–14]. Hsp90 function from all four loci is specifically inhibited by compounds like geldanamycin or radicicol, which compete with ATP for the nucleotide-binding pocket, so disrupting Hsp90 activity [15,16]. Inhibition shifts the cells from protein folding or activation, to degradation of most, but not all, unfolded client proteins by the proteasome system [17–20]. Complete loss of Hsp90 function is lethal, as multiple essential pathways are inactivated, a feature exploited in cancer treatment, where tumour-cell growth is heavily dependent on mutated oncogenic proteins that require Hsp90 assistance to function during malignant progression (reviewed in [21] and [22]). Increased binding affinity of Hsp90 inhibitors to these mutated protein complexes preferentially sensitises them to Hsp90 inhibition, allowing differential killing of malignant versus normal cells [23]. Use of Hsp90 inhibitors has also been proposed for amelioration of protein-aggregation-associated neurodegenerative diseases [24,25]. Hsp90 protein function is required at all times in eukaryotic cells [26,27], but under stress conditions higher levels are achieved through induction of a heat shock response. Recent genome-wide approaches have expanded the set of Hsp90 client proteins and required cofactors that are thought to confer substrate specificity [28–30]. These new findings are extending the role of Hsp90 from protein folding to gene regulation (reviewed in [31]) with potential impact on cellular epigenetic states (reviewed in [32]). Hsp90 inhibitors used above a threshold level induce a heat shock response through the activation of heat shock factor 1, which is normally silenced by association with Hsp90 [33].

Classical examples of developmental anomalies with variable severity and penetrance include holoprosencephaly, neural tube defects, cleft lip and palate, and heart malformations. Recently our human disease interests have focused on severe eye malformations, such as anophthalmia, microphthalmia, and coloboma [34–36]. These provide additional good examples of non-Mendelian inheritance patterns, where the relative contribution of environmental and genetic factors to the complex phenotypes remains controversial. If vertebrate phenotypes are also modulated by Hsp90, these could provide the molecular link between periodic environmental fluctuation and the overt manifestation of genetic susceptibility. If the chaperone system plays a buffering role in the variability of certain mutant phenotypes, compromising Hsp90 function may alter the severity or penetrance of some defined traits without affecting overall development.

Here we report that previously unobserved specific developmental anomalies were uncovered following partial inhibition of Hsp90 function in zebrafish. The spectrum of phenotypes observed and the organ systems affected depend both on the strain used and on the developmental stage at which the carefully controlled Hsp90 inhibitor is administered. Breeding studies clearly indicate that the phenotypes are manifestations of underlying cryptic genetic variation. Under our conditions, WT strains do not give rise to the rare malformations we observed in some mutant strains but do reveal more common anomalies that may reflect the uncovering of generally silent common gene variants. In addition to the de novo exposure of cryptic variation, we also showed that Hsp90 inhibition can modify the severity of some, but not all, already characterized developmental anomalies. Phenotype amelioration was observed in one case and increased severity in another. Both of the modified phenotypes were characterized as missense mutations at different loci. No phenotype modulation was observed at two different loci where the mutations had been identified as nonsense changes. Molecular studies show that in the presence of Hsp90 inhibition one of the mutant proteins studied is rapidly decreased, while its WT allele is largely unaffected, providing a molecular model for Hsp90 modulation of phenotype at the organismal level.

## Results

### Establishing Drug, Morpholino Doses, and Treatment Timing in WT Strains

Geldanamycin (GA) has been used previously to examine the requirement for Hsp90 during zebrafish development [37]. The drug dose used (35 μM GA) led to developmental malformations in 80% of the treated embryos regardless of the strains tested [37], and therefore independently of underlying genetic background.

To investigate treatment conditions that could affect the phenotypic outcome selectively in a strain-specific manner, we initially assessed the developmental stages and highest drug doses at which Hsp90 function is decreased without affecting viability, or inducing highly penetrant or severe developmental defects in WT strains (Table 1). Hsp90 function was reduced by treating embryos with radicicol (a macrolactone), which is functionally equivalent to but structurally distinct from GA (a benzoquinone ansanamycin). Subsequently we also used the GA class inhibitor 17-allylamino-17-demethoxygeldanamycin (17AAG) [38], which has low toxicity and is now in clinical trials for cancer therapy [21]. Morpholinos, for knocking down the activity of the two cytoplasmic forms of Hsp90 alpha (hsp90a *[Danio rerio]* and HSP90AA1 *[Homo sapiens]*) and Hsp90 beta (hsp90ab1 *[D. rerio]* and HSP90AB1 *[H. sapiens]*), were also used as additional controls for drug-specific inhibition of Hsp90.

**Table 1:**
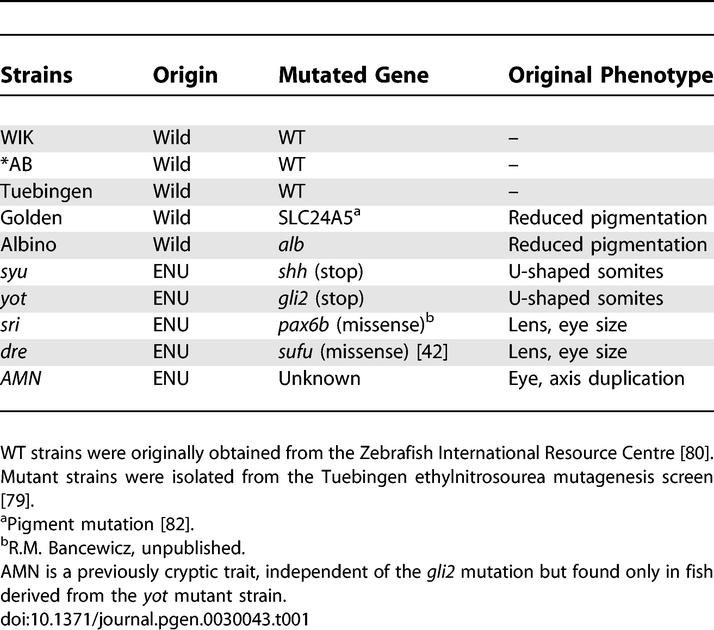
Zebrafish Strains Used in This Study

**Figure 1:**
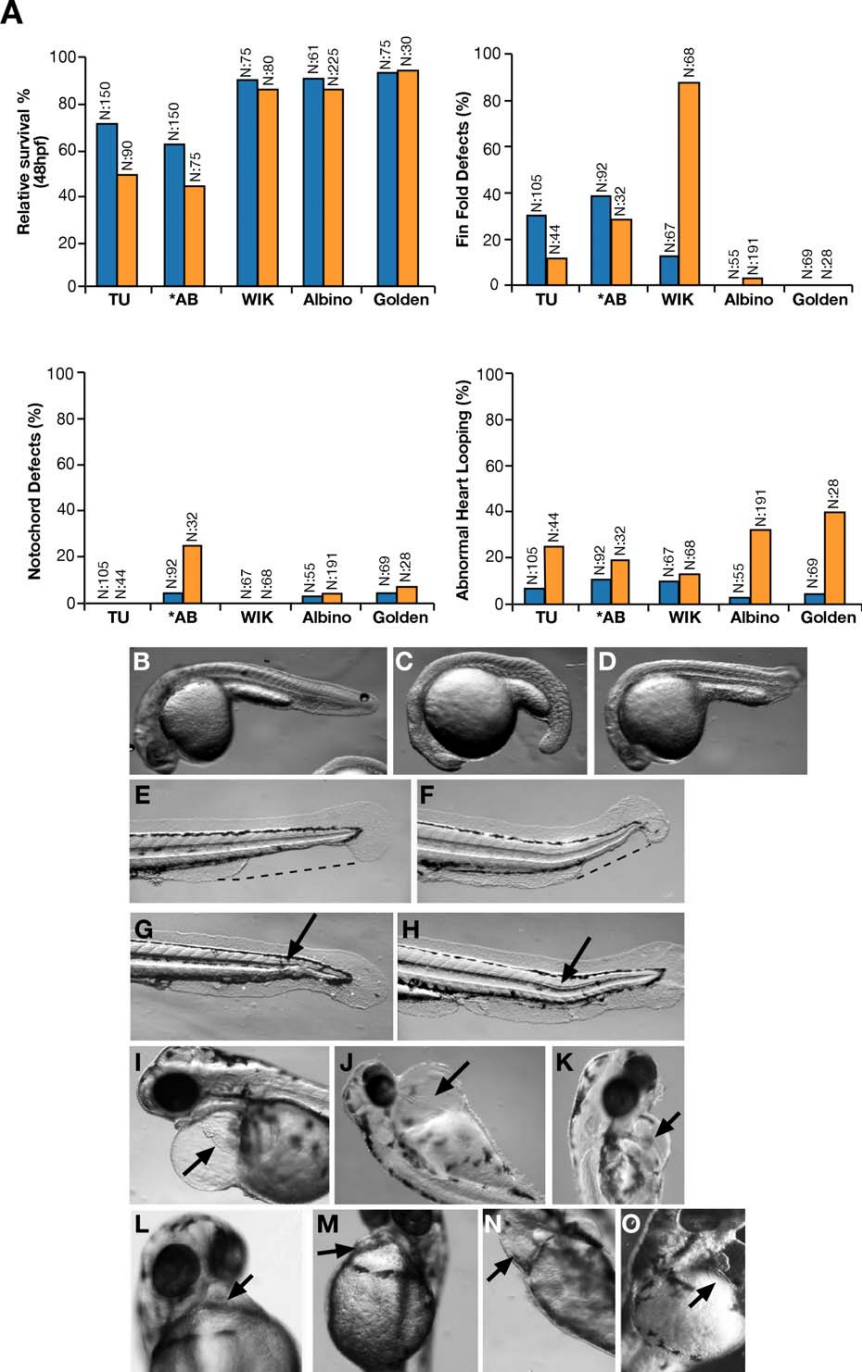
Developmental Defects Observed among WT Strains after Decreasing Hsp90 Function at 30% Epiboly (A) Frequencies: Embryos were treated at 30% epiboly with 10 uM radicicol (blue) or 3.3 μM 17AAG (orange). Rate of survival is the relative percentage of live embryos at 48 hpf compared to the relevant control siblings. Abnormal heart looping includes heterotaxia and nonlooping of the heart tube; severely oedematous fish with consequently abnormal heart were excluded. (B–O) Examples of observed phenotypes: (B–D) Developmental delay observed at 24 hpf after treatments. Interrupted caudal fin fold (dotted lines) after radicicol (E) or 17AAG (F) treatment. Notochord folds (arrows) after radicicol (G) and 17AAG (H). Drug-induced heart oedema with abnormal heart morphology after radicicol (I) and 17AAG (J). Radicicol-induced mild oedema with abnormal heart looping (K). WT (L) or inverted heart looping after 17AAG (M), radicicol (N), or Hsp90 morpholino injections (O). Black arrows indicate the position of the ventricle.

We tested a range of doses at 30% and 50% epiboly and recorded novel phenotypes and viability. When treatment was initiated at 30% epiboly (defined as 4.6 h postfertilization [hpf]), both drugs caused multiple phenotypes and reduced viability assessed at 24–48 hpf. For example, among those surviving to 24 hpf following treatment with 10 μM radicicol or 3.3 μM 17AAG, we repeatedly observed growth retardation, decreased pigmentation, pericardial oedema with or without abnormal heart morphology, fin-fold, and notochord defects (Figure 1B–1K). Both drugs caused mild oedema and rarely severe oedema with altered heart morphology (Figure 1I and 1J; Video S1). 17AAG also elicited inversion of left-right heart looping or heterotaxia in very high frequencies (Figure 1M; Videos S2 and S3), while after radicicol treatment only a few embryos displayed abnormal heart looping in the absence of oedema (Figure 1N, Videos S4–S6). Radicicol also produced a few cases of severe growth retardation. Most of these phenotypes were present in all WT strains tested, but with clearly different frequencies (Figure 1A).

**Figure 2:**
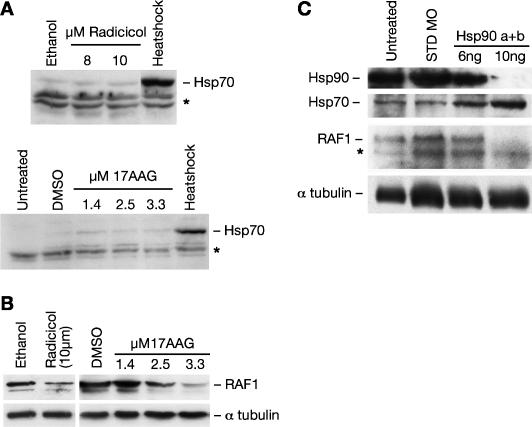
Assessment of Heat Shock Induction and Levels of Hsp90 Inhibition (A) Western blots to assess Hsp70 induction following overnight treatment of WT embryos initiated at 50% epiboly with increasing concentrations of radicicol or 17AAG. As positive control, Hsp70 was induced by incubating embryos at 37 °C for 30–60 min before harvesting. (B) Western blots showing graded reduction in RAF1, an Hsp90 client-protein, in response to inhibition by radicicol and 17AAG. (C) Western blots to assess Hsp90, Hsp70, and RAF1 levels in embryos injected with 6 ng of universal morpholino control (STD MO) or Hsp90 a+b morpholinos at the indicated individual amounts. α-tubulin was used as loading control. *, Nonspecific band showing equal loading

Higher survival rates and lower frequencies of developmental malformations were achieved by initiating treatment with the same doses but later in development, 50% epiboly (5.25 hpf). Many of the elicited phenotypes were milder and transient, like growth delay, fin-fold defects, or mild heart oedemas mostly with normal morphology and circulation. These embryos were generally indistinguishable from sibling controls by 5 d postfertilization (dpf). Notochord folds were noted but heart looping was unaffected. These phenotypes were again shared by most strains tested, albeit at slightly different frequencies. The penetrance of these common phenotypes in each strain was apparently decreased compared to earlier treatment (Figure S1). In agreement, these drug doses had no adverse developmental effects when treatment was initiated at 75% epiboly (8 hpf). We assessed the molecular events elicited by initiating treatment at 50% epiboly with the indicated drug doses by monitoring heat shock protein 70 (Hsp70) levels in treated embryos. Hsp70 was not induced at any of the inhibitor doses tested (Figure 2A). Hsp90 functional reduction was confirmed by monitoring the Hsp90 client protein RAF1, whose levels decrease with Hsp90 inhibition in a dose-dependent manner (Figure 2B) [39]. Although this gives an indirect estimation of Hsp90 inhibition, the residual presence of RAF1 protein, even at the highest inhibitor dose used, demonstrates that only partial Hsp90 functional reduction was elicited.

Morpholinos are injected at two to four cell stage, which decreases Hsp90 protein levels at earlier developmental stages than the selected conditions for chemical treatment; but doses that do not give rise to severe phenotypes when injected into WT embryos still help to validate chemical inhibition studies. Injecting the double morpholino knockdown (Hsp90 a+b: 6 ng of hsp90a and 6 ng of hsp90b) partially decreased Hsp90 protein levels (Figure 2C) and had no noticeable effect on embryos derived from the WT strains Golden (*n* = 71). Higher doses occasionally produced heart defects, such as inverted looping (Golden: 4/100) (Figure 1O) or no looping (WIK: 5/36) (Figure S2A), oedema (Golden: 10/100 and WIK: 0/36), and notochord defects (Golden: 2/100 and WIK: 0/36), but also decreased viability (Golden: 40/100). The low frequency of heterotaxia is in agreement with our observation that trait penetrance decreases when Hsp90 function is chemically reduced before 30% epiboly (unpublished data). Doses above 10 ng of each morpholino (Hsp90 a+b) almost completely erased Hsp90 protein, greatly reducing viability, with severe oedema in most embryos (17/20 injected) (Figure S2B). While the specific chemical inhibitors reduce the functional capacity of Hsp90 without decreasing Hsp90 protein levels, in morpholino-injected embryos Hsp90 levels were reduced in a dose-dependent manner with the consequent Hsp70 induction and decreased RAF1 levels (Figure 2C).

The two chemically unrelated drugs and morpholino knock down produced an overlapping spectrum of phenotypes, showing different frequencies and severities that may be due to differences in drug metabolism (between radicicol and 17AAG) [40–41], or treatment timing (between drugs and morpholino). Most of the phenotypes observed by initiating drug treatment at 30% epiboly were shared by all strains tested but revealed at different frequencies and therefore represent Hsp90 buffering of common gene variants or polymorphisms presumably present in different strain specific combinations. Treatment timing was critical for the penetrance of these traits.

Importantly, when drug treatment was initiated at 50% epiboly, rather than 30%, there was no significant impairment of development in embryos derived from WT strains. The selected drug concentrations reduce Hsp90 functional activity without inducing a heat shock response, which could obscure the effect of Hsp90 reduction during development. Consequently, to investigate the effect of Hsp90 phenotype modulation on genetic mutants, we treated embryos at 50% epiboly with doses of 10 μM radicicol or 3.3 μM 17AAG, or lower, so minimizing the potential appearance of common developmental defects by narrowing the developmental window for the perturbation.

### Hsp90 Modifies the Severity of Mendelian Eye Mutants

Variable severity, age of onset, and intrafamilial variability are often observed in classical Mendelian monogenic diseases. In order to investigate possible factors that could alter the phenotypic outcome of simple traits, we assessed the effect of partial Hsp90 inhibition on two Mendelian eye mutants: *sunrise (sri)* and *dreumes (dre)* (Table 1).

**Figure 3:**
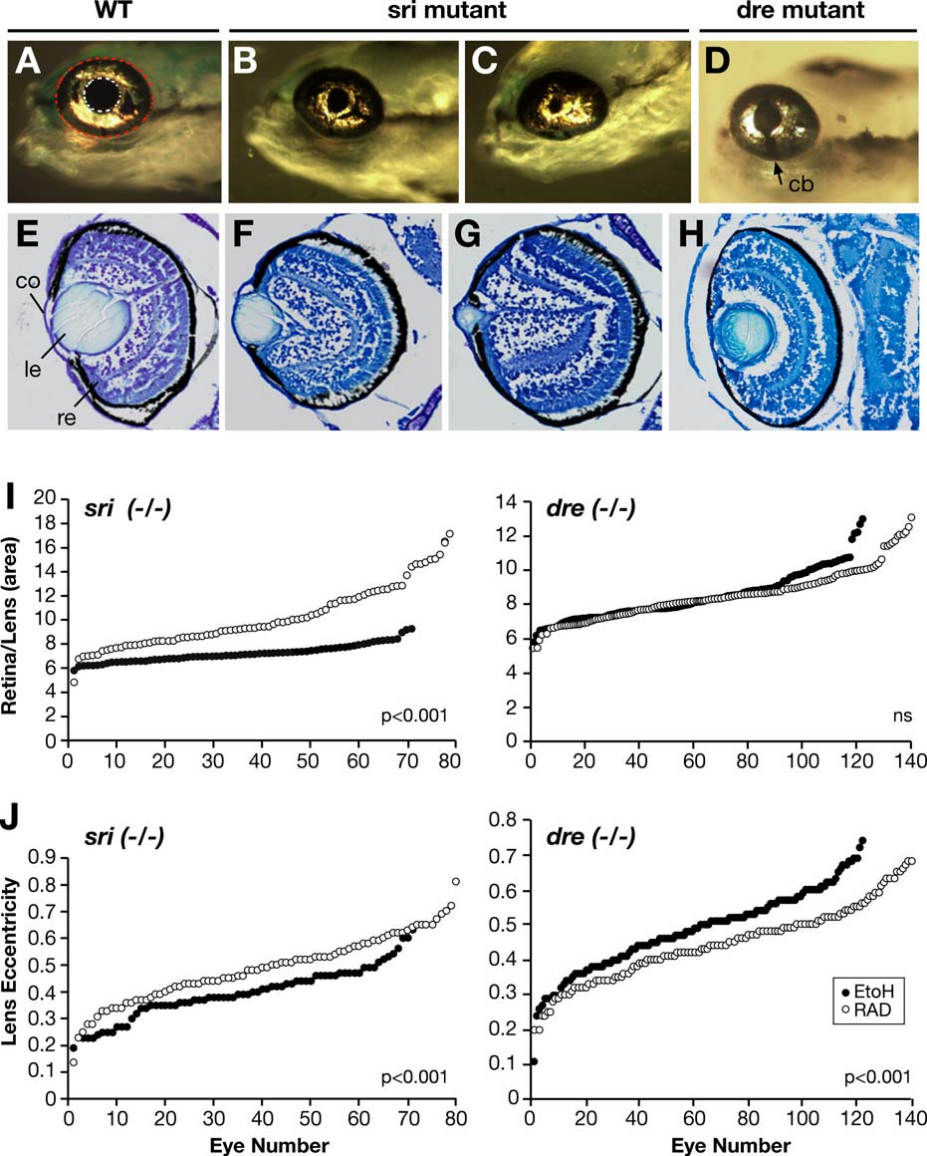
Assessing Range of Expressivity and Quantifying Phenotype Modulation in *sri* and *dre* Strains (A–H) Phenotype of the *sri* and *dre* eye mutants at 5 dpf. (A–D) Lateral view of live embryos; (E–H) histological sections. (A) WT; lens (white-dotted lines) and retina (red-dotted lines) measurements were used to asses phenotypic severity; (E) WT section showing retina (re), lens (le), cornea (co); (B) and (F) *sri* mild and (C) and (G) *sri* severe; (D) *dre* with coloboma (cb); (H) *dre* showing flattened anterior segment. (I and J) Ranked plots showing distribution of quantitative eye parameters in treated (white circles) and sibling control (black circles) homozygous embryos: (I) Ratio of retina to lens area; (J) Lens shape or eccentricity.

The *pax6b* mutant *sri* (R.M. Bancewicz, unpublished data) and the *sufu* mutant *dre* (Table 1) [42] both show abnormal lens development (Figure 3) [43], with complete penetrance but variable severity [1]. This is demonstrated by a decrease in lens size relative to the retina (Figure 3B and 3C) reflecting abnormal development of surface ectoderm-derived structures [44]. Occasional failure to close the optic fissure (coloboma) is also observed (Figure 3D). Upon Hsp90 functional reduction, lens size relative to the area of the retina was significantly decreased in homozygous *sri* embryos while homozygous *dre* remained unaltered (Figure 3I; Table 2). Lens shape was significantly altered in both homozygous mutants (Figure 3J; Table 2); but while in treated *sri* embryos the lens became more elliptical, in treated *dre* embryos it became more circular, more closely resembling lens shape in WT fish. A 3-fold increase in the frequency of colobomas was also observed among treated homozygous *sri* embryos (*p* < 0.021), while in treated *dre* embryos a slight decrease was seen (1.6% versus 0.4%). These results show that decrease in Hsp90 functional capacity can significantly influence the expressivity of Mendelian traits in a way that is clearly dependent on the genetic background.

**Table 2:**
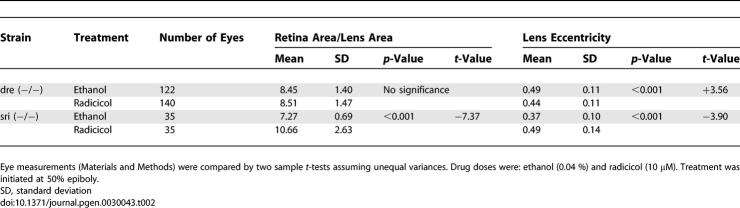
Quantitative Effect of Hsp90 Inhibition on Embryos Derived from Eye Mutants

The significant effect of Hsp90 inhibition on the *sri* background prompted us to investigate whether dominance relationships could be altered in vertebrates, as previously observed in *Drosophila* and *Arabidopsis* [7]. Notably, in heterozygous *sri* embryos and in an additional genetically unrelated strain (AMN strain) (Table 1), these parameters were unaffected by the drug treatment (Table 3). The different susceptibility of homozygous and heterozygous *sri* embryos demonstrates that the increased phenotypic severity in homozygotes is due to the synergistic effect between decreased Hsp90 function and the homozygous mutant phenotype rather than to additive effects between reduced Hsp90 function and increased dose of the mutant Pax6b protein.

**Table 3:**
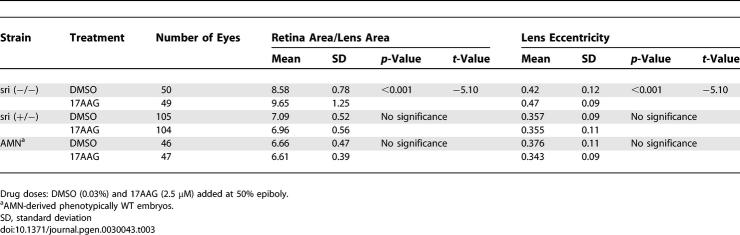
Synergistic Effect of HSP90 Inhibition and Homozygous *sri* Phenotype

### Factors That Contribute to Phenotype Modification by Hsp90 Inhibition

The observation that both lens size and shape can be affected in *sri* derived embryos but only lens shape was altered in *dre* embryos suggests that the two developmental processes can be uncoupled. As described above, Hsp90 chemical inhibition by the structurally distinct drugs radicicol or 17AAG significantly reduced lens size and worsened lens eccentricity of *sri* homozygous embryos (Table 4). Geldampicin (GMP), a structurally related but inactive form of 17AAG, was used as a negative control to discount direct drug toxicity as the mechanism for this phenotype modulation [45]. GMP had no effect on these parameters (Table 4). We then decreased Hsp90 activity with the combined morpholinos: Hsp90 a+b. Coinjecting 6 ng of each morpholino significantly affected lens size without altering lens shape (Table 4), confirming that the two developmental processes can be uncoupled. Hsp90 can also be diverted from its developmental role by the induction of a generalised stress response [46]. Brief heat exposure of homozygous *sri* mutants also resulted in altered phenotypic severity not observed in control siblings (Table 4). Interestingly, lens size and shape were affected by heat shock, but in contrast to Hsp90 chemical inhibition, lens shape was ameliorated instead of worsened (compare *t*-values, Table 4). Several factors may be contributing to these phenotypic outcomes: functional reduction of Hsp90 by these treatments may be milder than Hsp90-specific chemical inhibition. This seems likely as RAF1 levels observed after morpholino injections are comparable to those seen in control embryos, despite the reduced Hsp90 protein levels (6 ng Hsp90 a+b, Figure 2C). Higher morpholino doses drastically reduced RAF1 levels but mostly produced severely oedematous embryos whose eyes could not be analysed. More significantly, we observed a positive correlation between Hsp70 levels and amelioration of the lens eccentricity (6 ng Hsp90 a+b, Figure 2C; Hsp70 levels, Table 4). Hsp70 is constitutively expressed during normal lens development from 28 hpf [47]. Earlier induction by heat treatment or morpholino knock down may contribute to the partial rescue of the *sri* lens phenotype, in contrast to drug treatment conditions that were specifically selected to avoid inducing a heat shock response. Heat shock also induces Hsp90 expression, and this may contribute additional capacity to cope with the increased requirement for chaperone activity following heat shock.

**Table 4:**
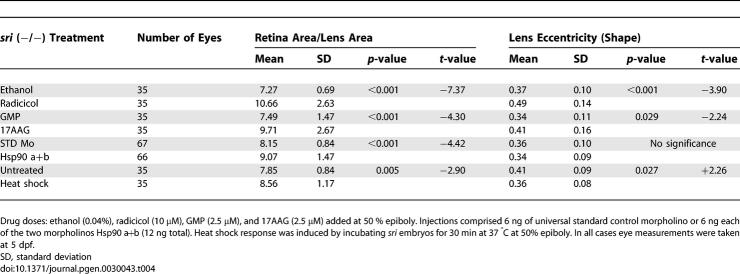
Morphological and Quantitative Components Are Differentially Buffered by Hsp90

### Mendelian Mutants Resistant to Mild Hsp90 Functional Reduction

To address how commonly Hsp90-mediated release of genetic variation is observed, we selected Mendelian mutants that affect somitogenesis, a developmental process previously found to require the chaperone function of Hsp90 [37,48]. We used the same experimental conditions as for the eye mutants.

We studied the semidominant *you-too (yot)* mutant, with U-shaped somites, carrying a premature protein truncation mutation in the *gli2* gene (Table 1) [49]. Although live heterozygous *yot* embryos are indistinguishable from wild type, histological analysis had shown a reduction in the MyoD expression [50]. Murine MyoD has been previously identified as an Hsp90 client protein [51], which dissociates from the cochaperone cdc37 in the presence of GA potentially interfering with myoblast differentiation programs [52]. The homozygous *yot* phenotype is fully penetrant with variable severity. Hsp90 inhibition did not significantly worsen the homozygous phenotype of *yot* embryos (unpublished data), nor were somite defects uncovered when heterozygotes were treated with radicicol. Thus Hsp90 functional reduction had no effect on the gli2 pathway in spite of the already compromised low levels of MyoD protein.

The phenotypically variable, recessive somite mutant *sonic-you (syu)* with a stop codon mutation in the *sonic hedgehog* gene *shh* [53] was also unaffected by Hsp90 inhibition (unpublished data). Thus, mild perturbation of Hsp90 function does not affect all Mendelian phenotypes during vertebrate development emphasizing the selective interplay between Hsp90 buffering and the underlying genotype.

### Hsp90 Functional Reduction Reveals Previously Cryptic Eye Trait

**Figure 4:**
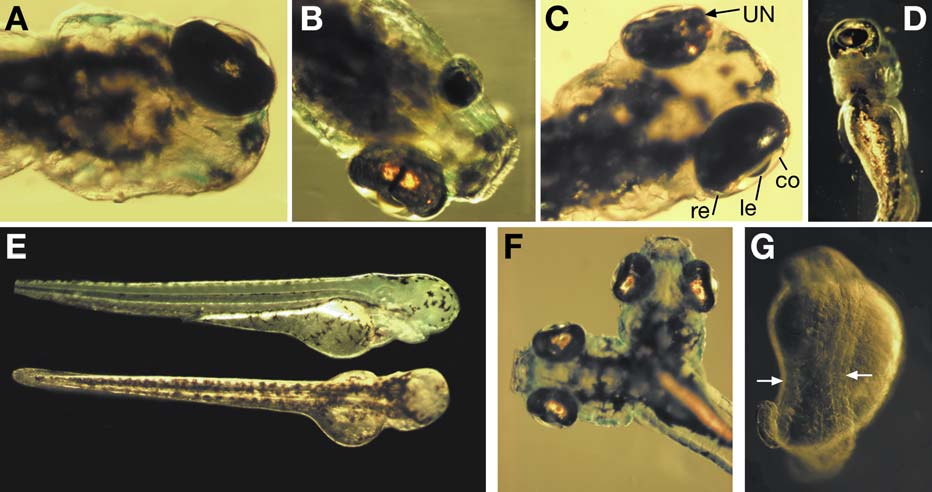
Eye Defects and Body-Axis Duplications Observed in AMN Population Dorsal views shown are: (A) Unilateral anophthalmia; (B) Unilateral microphthalmia; and (C) Unilateral nanophthalmia (UN), illustrating retina (re), lens (le), cornea (co). (D) Ventral view of cyclopic embryo is shown. (E) Bilateral anophthalmia is shown (top, lateral view; bottom, dorsal view). (F) Anterior body-axis duplication (ABD), two complete heads formed; incomplete ABD were also observed (not shown). (G) Posterior body duplication (PBD), two notochords showing adjacent somite boundaries (arrows), culminating in two tails.

During our studies of the *yot* mutant strain, radicicol treatment revealed novel eye phenotypes not previously described in heterozygous or homozygous embryos of this strain [54,55]. These new phenotypes, ranging from bilateral anophthalmia to unilateral microphthalmia (Figure 4), were never observed in three other tested mutants, *syu, dre,* or *sri* (Table 1). Molecular analysis proved these eye defects to be independent of the *gli2* mutation in the *yot* strain, and the *yot* genotype and phenotype were rapidly lost from carriers of this new variable eye trait, named AMN for anophthalmia, microphthalmia, and nanophthalmia.

**Figure 5:**
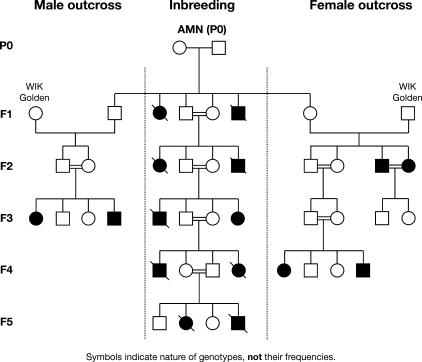
Inheritance Patterns of AMN AMN parental carriers were crossed and the subsequent generations raised following inbreeding or outcrossing to genetically unrelated WT mates (white symbols, phenotypically WT fish; black symbols, AMN affected fish; squares, males; circles, females). Line across black symbol indicates lethality of affected phenotype. Outcrosses showed two types of transmission: maternally determined (female outcross) and zygotically determined (male outcross) trait. After inbreeding, only WT embryos were viable. After outbreeding, some affected embryos were viable and fertile. The mating of affected fish produced only WT embryos illustrating the requirement for genomic homozygosity at some locus/loci in the oocyte (female outcross). Most carrier pairs transmitted the trait at a frequency of 0.5%–5%. For the few high-transmitting parents, the frequency of affected embryos without treatment was never greater than 13% in any one cross.

In seven *yot-*derived mated pairs, from a total of 24, partial Hsp90 inhibition repeatedly gave rise to affected offspring; these were designated transmitter parents for the AMN eye trait (P_0_, Figure 5; Table 5). Their offspring revealed a significant increase in the frequency of developmental eye phenotypes compared to those from nontransmitter pairs and from genetically unrelated WT strains (Table 5). AMN-like defects were not induced repeatedly in any other mutant or in WT strains tested (*n* > 3,000), where even higher inhibitor doses (10 μM radicicol or 3.3 μM 17AAG and Hsp90 morpholinos) or earlier treatment (at 30% epiboly) were ineffective (Multiple Unrelated Strains, Table 5; unpublished data). Except for two embryos among several thousand scored, whenever eyes were affected in WT-treated stocks, it was in the presence of other more severe defects like oedema or incomplete development. These occasional abnormal phenotypes do not recur in subsequent generations and their frequency was unaltered by Hsp90 inhibition; they were not counted in the analysis (Multiple Unrelated Strains, Table 5). Where reduced Hsp90 function repeatedly reveals increased frequency of offspring with a particular phenotype, it is deemed to be uncovering a previously cryptic mutation in susceptible populations (Parental Transmitters, Table 5). Unlike abnormal heart looping, notochord, or fin-fold defects, the AMN phenotype was revealed uniquely in the *yot* population. Hsp90 inhibition therefore uncovers cryptic phenotypes selectively in susceptible populations, during vertebrate development.

**Table 5:**
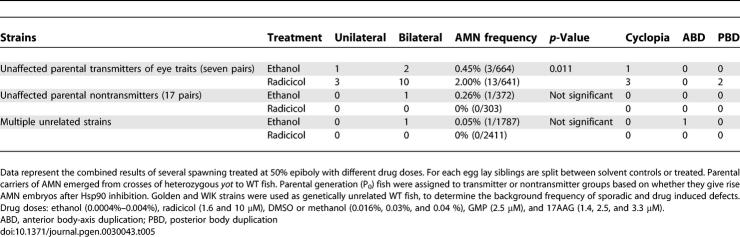
Frequency of Eye and Body-Axis Defects in Parental AMN and WT Strains

### Complex Inheritance Patterns of Cryptic Trait

The low viability of AMN-affected embryos means that most of the offspring raised for subsequent breeding were phenotypically WT. From these, 11 females were crossed to 15 males in 21 different combinations. Half of the pairs transmitted the AMN trait, albeit at greatly different frequencies (0.5%–13%) in the absence of Hsp90 inhibition. This suggests a genetic basis involving several loci, some of which are required to be homozygous. Highly variable frequencies of affected offspring in different batches of eggs from the same mating pair demonstrate the variable penetrance of the trait. Additional rare phenotypes like cyclopia (fused central eye) (Figure 4D; Tables 5 and 6) and posterior body-axis duplications (Figure 4G) reappeared after inbreeding (Table 6). Some offspring showed novel phenotypes such as nanophthalmia (small eyes with normal structure) and anterior body-axis duplication (Figure 4C and 4F), indicating that different recessive interacting loci are brought together at an early stage following inbreeding. The almost continuous range of phenotypes conforms to a threshold model [56] where several modifiers are required to interact genetically with a primary recessive mutation to elicit the phenotype [57]. The complexity of the gene interactions will be further illustrated by divergent responses to Hsp90 inhibition (see below).

**Table 6:**
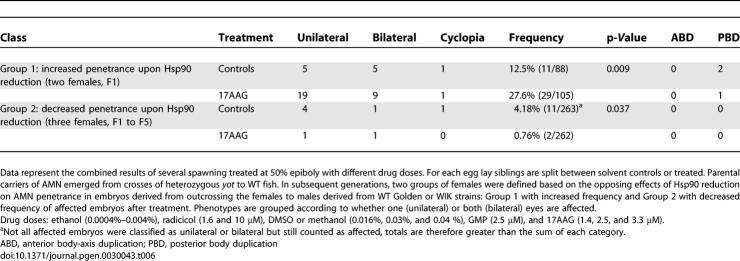
Two Classes of AMN Transmitter Females Identified

The inbreeding regime maintained the complex eye trait for at least five generations but never fixed it at Mendelian frequencies, most likely because only phenotypically unaffected individuals could be mated (Inbreeding, Figure 5). The low frequency of AMN defects observed in subsequent generations is another indication of the oligogenic nature of the trait. Nevertheless, the sensitivity to reduced Hsp90 levels remains. Female transmission of the AMN trait was observed after outcrossing phenotypically WT F1 carrier females to genetically unrelated WT males (F2, Female Outcross, Figure 5) with variable frequencies. The affected individuals derived from these outcrosses were viable and fertile, suggesting loss of some deleterious modifiers. However, the phenotype was not observed in the generation arising from these affected sibling crosses (F3, Female Outcross, Figure 5), but it was recovered after further inbreeding (F4, Female Outcross, Figure 5). This inheritance pattern is consistent with a requirement for a maternal recessive component with partial penetrance. In contrast, when selected males were outcrossed to genetically unrelated WT females, AMN-like embryos were not observed initially (F2, Figure 5, Male Outcross). However, AMN-like embryos were recovered in the first generation of subsequent inbreeding (F3, Male Outcross, Figure 5) suggesting that homozygosity of a recessive zygotic component is required. The recessive genetic basis of the AMN trait, suggested by these inheritance patterns, does not exclude the possibility that additional genetic or epigenetic modifiers also affect the severity and/or penetrance of the AMN phenotype.

### Hsp90 Inhibition Reveals Digenic Nature of the AMN Trait

**Figure 6:**
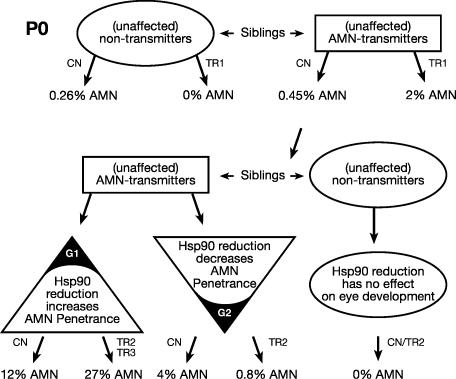
Schematic Representation of AMN Phenotype Inheritance and Susceptibility to Hsp90 Reduction Embryos were treated with the solvent control (CN) or with radicicol (TR1), 17AAG (TR2) or Hsp90 a+b morpholino injection (TR3). Following Hsp90 inhibition, embryos derived from group 1 (G1) females showed increased penetrance, while those from Group 2 females (G2) had decreased penetrance of AMN phenotype.

Although all transmitting females tested produce some affected offspring upon outcrossing to an unrelated WT male, treatment of embryos from these pairs revealed two types of AMN transmitter females: Two inbred F1 females crossed to four different WT males showed a significant increase in frequency of AMN offspring when embryos were treated with 17AAG (Figure 6; Group 1, Table 6). In contrast, three inbred females from F1 to F5 crossed to seven different males produced fewer AMN-affected embryos with 17AAG treatment than without (Figure 6; Group 2, Table 6). Both Group 1 and male transmission frequencies of AMN are also increased when Hsp90 inhibition is elicited by morpholino injections knocking down hsp90a and hsp90b (*n* injected = 154; frequency, 10%; *p* = 0.02). Once more, these results demonstrate that partial inhibition of Hsp90 function can increase as well as decrease phenotypic penetrance of disease traits and further suggest that at least two segregating loci are involved in the original AMN phenotype.

### Components of the Canonical Wnt Pathway May Underlie the AMN Complex Trait

**Figure 7:**
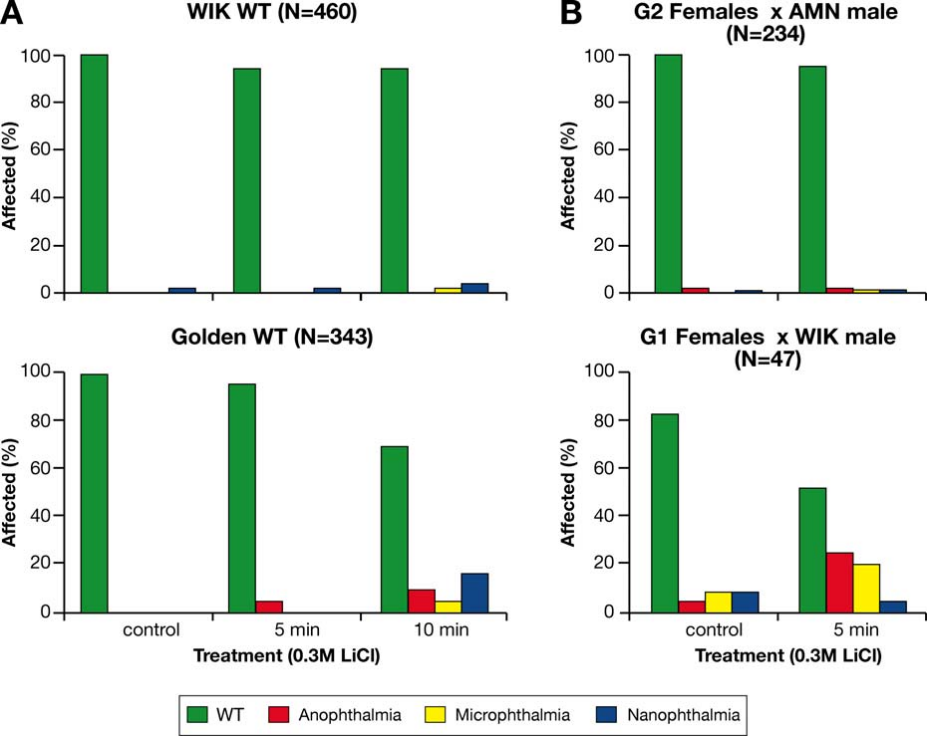
Sensitivity of Group 1 Embryos to Lithium Chloride: An Inducer of Wnt Signalling (A) Assessing duration of LiCl treatment that would not significantly affect embryos from WIK and Golden strains. (B) LiCl treatment (0.3 M for 5 min) of embryos from Group 1 females outcrossed to WIK males gave rise to increased frequency of eye defects, while embryos derived from Group 2 females crossed to WT (not shown) or AMN transmitting males did not.

Anterior and posterior body duplications were also observed in the offspring of Group 1, but not Group 2 females. The copresentation of axis duplication with cyclopia or absent eyes is reminiscent of phenotypes caused by alterations in the canonical Wnt signalling pathway [58–60]. To test this suggestion, embryos derived from both groups of females were treated for five minutes with lithium chloride, a glycogen synthase kinase 3b inhibitor that mimics Wnt signalling [59,61]. At the selected doses and incubation times, embryos derived from WT strains, or from Group 2 females incrossed to AMN transmitting males, were barely affected (Figure 7) [59]. In contrast, lithium-treated embryos from Group 1 females outcrossed to genetically unrelated WT males show a significant increase in the number of eye defects compared to their untreated siblings (*n* = 47; *p* = 0.001) (Figure 7). Genes from the canonical Wnt pathway may, therefore, be implicated in the genesis of the eye trait in Group 1.

In agreement with the contrasting phenotypic outcomes following Hsp90 inhibition in embryos derived from Group 1 and Group 2 females, these results indicate that the original AMN trait comprises at least two loci sensitive to Hsp90 fluctuation. Recombining of variant alleles at these seemingly antagonistic loci may explain the sudden rise in the frequency of AMN from parental to F1 generation, observed in some of the crosses.

### Modulation of Developmental Stability in Vertebrates by Hsp90

Symmetry of bilateral traits is often used as a parameter for developmental stability [62]. Measures of developmental instability include nondirectional quantitative deviation from bilateral symmetry, also called fluctuating asymmetry. Other asymmetric manifestations include unilateral absence of organ structures and perturbed organ distribution across the left-right axis or phenodeviants [63].

We observed a relative increase in the frequency of unilaterally affected eyes (mostly anophthalmia) in embryos from AMN females following Hsp90 functional reduction by inhibitor treatment or morpholino knock down (Table 7). Similarly, we observed a significant increase in the number of unilateral colobomas among *sri-*treated embryos (Table 7) The relative increase in the frequency of unilateral morphogenic events between controls and treated embryos, suggests that Hsp90 can buffer developmental stability in vertebrates.

**Table 7:**
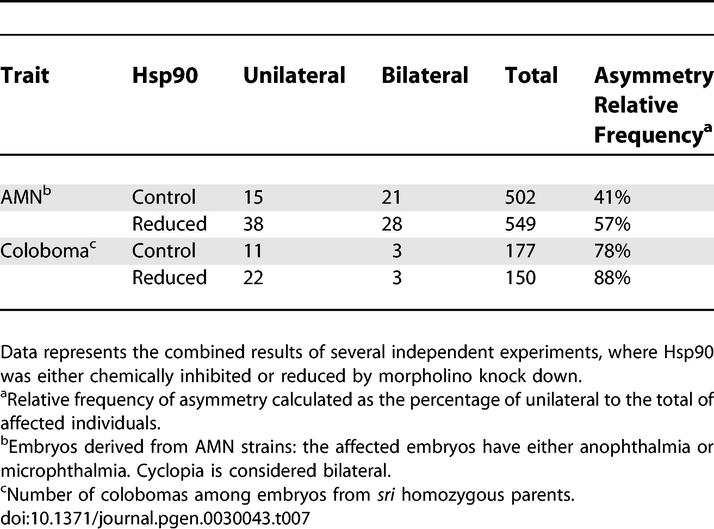
Compromised Hsp90 Function Increases the Relative Frequency of Discrete Asymmetric Individuals

**Figure 8:**
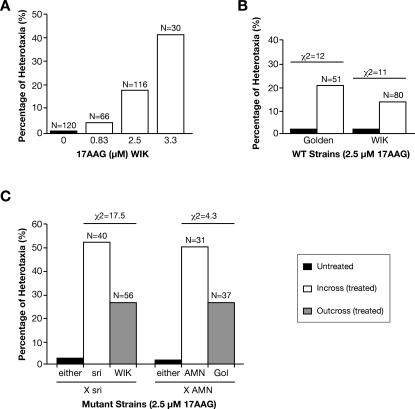
Inversion of the Left to Right Heart Looping among 17AAG-Treated Embryos (A) Dose-dependent induction of heterotaxia in WT strain WIK, following treatment at 30% epiboly. (B) Comparison of inversion frequencies observed in the WT strains Golden and WIK. (C) Inbred mutant lines (*sri* or AMN) show significantly increased frequency of heterotaxia compared to WT strains at the same drug dose. Outcrossing these lines to WIK or Golden decreased the frequency to almost WT levels.

Inverted cardiac looping could be considered as another indication of compromised homeostasis during development. When 17AAG treatment is initiated at 30% epiboly we observed a dose-dependent increase in the frequency of heart-loop reversal or heterotaxia in WT strains (Figure 8A). Interestingly, at the same dose (2.5 μM 17AAG) the frequency of induced heterotaxias is higher in embryos from mutant strains like *sri* or AMN than in WT strains (incrosses, Figure 8B and 8C). When these carrier fish are outcrossed to WT strains, heterotaxia frequencies are significantly decreased (outcross, Figure 8C), suggesting that increased susceptibility to heterotaxia may be associated with homozygosity at predisposing loci. Alternatively, a previous unrelated mutation, which would not contribute to the trait under normal circumstances, could compromise development, rendering it more susceptible or dependent on fully functional buffering mechanisms like Hsp90.

Previous work has shown that in *Drosophila,* Hsp90 does not affect the fluctuating asymmetry of quantitative traits [64]. In contrast, Hsp90 can buffer stochastic processes in normal plant development [8]. Given the impact of Hsp90 reduction on the unilateral appearance of anophthalmia and coloboma among treated individuals over their sibling controls in some of our fish strains, we investigated whether Hsp90 could increase intraindividual quantitative differences in these mutants. Surprisingly, although Hsp90 reduction significantly affects the severity of lens defects in *sri* mutants (Table 3), it did not alter the variation between left and right measurements of any of these quantitative parameters (Table 8). Thus, as in *Drosophila,* under our experimental settings and statistical methods, Hsp90 plays no role on the developmental stability of quantitative traits. In contrast, Hsp90 clearly alters the consequences of stochastic processes on morphogenic or qualitative threshold traits like AMN, coloboma, and heart looping.

**Table 8:**
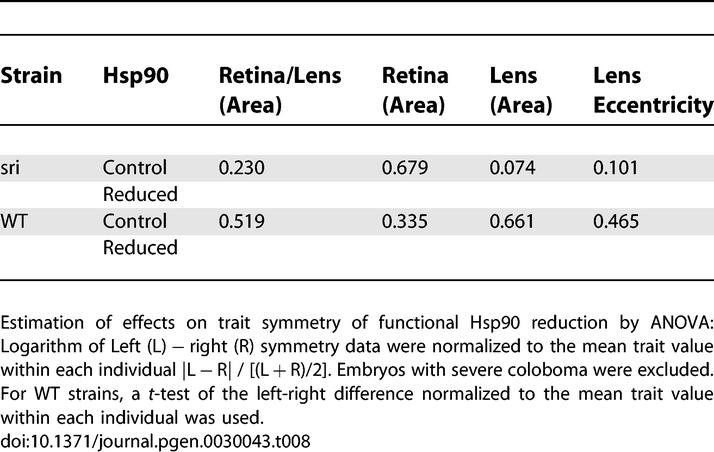
Compromised Hsp90 Function Does Not Affect Intraindividual Quantitative Differences (*p*-Values)

### Hsp90 Can Modulate Phenotypes by Selective Chaperoning of Mutant Proteins

**Figure 9:**
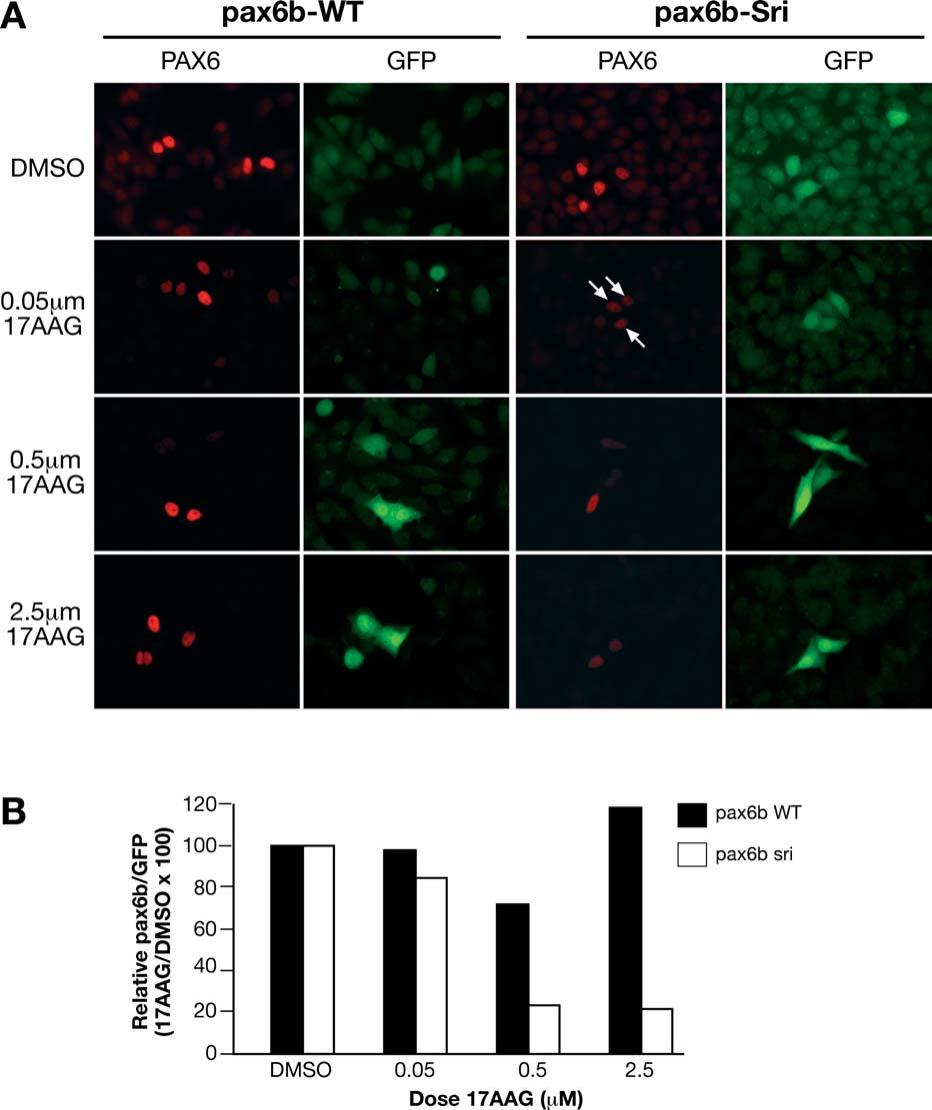
Differential Sensitivity of WT and *sri* Mutant Pax6b Protein to Hsp90 Inhibition Cells were cotransfected with either WT or *sri* mutant paxb cDNA and GFP reporter. (A) Representative field of HeLa cells transfected with Pax6b-WT or Pax6b-*sri* mutant immunohistochemically assessed for Pax6b protein levels (red) or control GFP frequency (green). Effects of treatment with different concentrations of 17AAG are shown compared with DMSO control. (B) Relative ratio of Pax6b positive (red cells) to GFP positive (green) cells for each treatment compared to the relevant DMSO control. The figures show one representative experiment from three independent experiments with similar results.

In order to understand phenotypic modulation of mutant phenotypes by Hsp90, we investigated the effect of compromised Hsp90 function on WT and *sri* mutant Pax6b proteins. Initial studies assessed the response of transfected WT and mutant expressed proteins to Hsp90 inhibitors. The *sri* mutation is expected to disrupt the folding of the homeodomain that contains it (R.M. Bancewicz, unpublished data). Immunohistochemical studies show that while WT Pax6b protein remains mostly unaffected, the levels of Pax6b mutant protein are significantly decreased when Hsp90 function is impaired by 17AAG treatment (Figure 9). The decreased stability of the mutant protein suggests that it requires Hsp90 for maintenance in a stable conformation. The differential sensitivity of mutant and WT proteins when Hsp90 function is compromised provides a molecular explanation for the worsening of the sri phenotype upon treatment. *pax6a* and *pax6b* in zebrafish are the paradigm model for subfunctionalisation by differential distribution of long-range control elements (D. Kleinjan, personal communication). The nonoverlapping expression pattern of the two Pax6 isoforms leads to the lens-specific phenotype of the *sri* mutant and to the worsening of this phenotype when chaperone function is inhibited.

## Discussion

Development is surprisingly robust to environmental, genetic, and stochastic variation. How the integration of multiple pathways, acting in narrow developmental windows, culminates in morphologically constant phenotypes is the subject of continuing debate. At the molecular level, genome duplication along with increasing complexity of epigenetic codes, chromatin regulation, and genomic organisation have led to increased compartmentation and subfunctionalisation, and to an improved capacity to absorb and buffer genetic variation. However, the underlying variation and phenotypic plasticity can be exposed by perturbing the buffering systems (reviewed in [65–66]). Modulating Hsp90 function is one mechanism that has been shown to reveal cryptic variation. Unravelling the mechanisms of variability in heritable phenotypes is important for understanding disease predisposition and penetrance. Here we show how vertebrate phenotype can be modulated by the chaperone Hsp90 and start to decipher the molecular underpinnings of this buffering mechanism.

Fine-tuning the titration and timing of Hsp90 functional reduction allowed us to demonstrate that Hsp90 is also able to buffer hidden genetic variation in vertebrates. The common phenotypes, revealed at different strain-specific frequencies in WT stocks, may represent common polymorphisms present in the available fish populations at several different possibly interacting loci, suggesting Hsp90 buffering may be responsible for canalising polygenic traits present in noninbred populations. Delaying treatment timing decreased the penetrance of these shared phenotypes and revealed the role of Hsp90 in rare malformations, now dependent on unique strain-specific mutations. Reducing Hsp90 function, within the relevant developmental window, generally increased the frequency of abnormal phenotypes such as anophthalmia and microphthalmia, and it altered the severity of some, but not all, Mendelian phenotypes studied. The resistance of some Mendelian mutants and the opposing (worsening versus amelioration) responses in those susceptible to Hsp90 fluctuation demonstrate that vertebrate Hsp90 buffering is a highly selective mechanism, modulating only defined genetic components of phenotypic variation. The recessive nature of the susceptible alleles characterised in this study suggests that the higher frequency and broader spectrum of phenotypes uncovered in *Drosophila* and *Arabidopsis* may be influenced by the relatively inbred nature of these organisms [5,8]. Zebrafish do not tolerate a high level of inbreeding, perhaps providing a better model for generally outbred human populations. The number of Hsp90 loci and the different spectrum of environmental triggers may also influence Hsp90 buffering capacity across phyla.

Opposite outcomes in the Mendelian microphthalmia mutants *sri* and *dre,* with the same tissues affected in both, demonstrate that Hsp90 modulation of phenotypes strongly depends upon the specific underlying genetic components. Despite the strong effect that Hsp90 inhibition had in *sri* homozygous embryos, it had no measurable effect in heterozygotes. This nonadditive response of the *sri* homozygous mutants to Hsp90 fluctuation confirms the role of Hsp90 in the maintenance of trait thresholds [67]. In contrast to plants and flies [7], these results imply that dominance relationships [1] may not be alterable in vertebrates by Hsp90—that is, previously recessive characters do not become dominant. The severity of the *sri* phenotype was also increased by heat shock. From the evolutionary point of view, increased temperature may not be a frequent mechanism through which Hsp90 releases variability in mammals, but this mechanism probably underlies the observations that maternal fever in humans and mild experimental hyperthermia in animals are associated with increased frequencies of developmental malformations, both mild and severe [68–70]. Similar adverse phenotypic outcomes may be elicited through induction of protein-folding stress in the developing embryo as a result of exposure to relatively low levels of environmental pollutants, or when endogenous protein aggregation levels are increased [71].

The uncovering of the complex AMN phenotypes focuses attention on the role of Hsp90 in oligogenic malformations. The involvement of multiple interacting loci, several with recessive effect, is indicated by the requirement for inbreeding, and the less than 25% peak frequency observed for AMN phenotypes. The involvement of two distinct sets of genes, but perhaps with overlapping functions, is suggested by the observation that AMN frequencies may be increased or decreased following Hsp90 inhibition in different parental groups. These opposing responses once more underline the complex mechanisms of interaction between developmental alleles and Hsp90-mediated release of genetic variation. The possibility of decreased phenotypic severity under stress conditions (Hsp90 reduction) is potentially of great evolutionary significance.

The rapid unmasking of divergent phenotypes after Hsp90 reduction helped us to break down the AMN trait into simpler components, indicating that this vertebrate model system may provide a robust approach to identifying cryptic genetic variation that can modify complex developmental traits. Similarly, heart-loop inversion was observed after earlier Hsp90 inhibition during development in all fish stocks tested, but the different frequencies among WT and mutant strains suggest underlying interacting genetic components. Further analysis of this may permit the identification of common susceptibility alleles at interacting loci. The observation that mutations at loci that would not normally contribute to a certain trait (e.g., *pax6b* in heart looping) may potentiate the effect of compromised Hsp90 buffering, suggests that Hsp90 may act in concert with the organism's genotype. This highlights once more the different release of cryptic genetic variation in good health and disease [72].

Our results show that in vertebrates, Hsp90 can buffer the phenotypic variability between and within individuals of discrete qualitative morphological traits (mainly anophthalmia and coloboma in this study) culminating in morphologically asymmetrical individuals. The increased frequency of asymmetric unilateral eye defects upon Hsp90 inhibition implies that buffering by this chaperone system contributes to the stabilization of developmental processes that depend on the partition of signalling molecules across body axes. Asymmetric phenotypes could result from stochastic uneven left/right partitioning of signalling molecules. Altered left-right embryonic signalling can affect heart looping in a simi-lar manner. It will be interesting to investigate how perturbation of Hsp90 activity amplifies the effect of these seemingly stochastic events, culminating in a significant increase of abnormal cases. The occurrence of unilateral and bilateral defects among AMN siblings is also strongly reminiscent of the complex human microphthalmia, anophthalmia, and coloboma spectrum [34] and indicates that environmental and stochastic factors continue to play an important role even when there are shared genetic components.

We show that in vertebrates, Hsp90 can also modulate phenotypic severity of continuous quantitative traits (e.g., relative lens size and lens shape in *sri* mutants). Surprisingly, Hsp90 inhibition does not alter small quantitative left-right differences of these traits, either in *sri* mutants or in WT strains. This raises the question of a fundamentally different molecular basis for developmental instability of discrete and quantitative (continuous) traits in vertebrates.

To date neither the identity of specific targets, nor the molecular mechanisms by which Hsp90 modulates phenotypic expression of genetic variants at the organismal level have been identified. The *sri pax6b* mutant allowed us to begin to unravel the molecular basis of Hsp90 buffering. The differential sensitivity of the Pax6b *sri* mutant protein in the presence of Hsp90 inhibitors suggests that Hsp90 chaperones the mutant protein; this may permit recovery of some functionality, thereby decreasing the phenotypic consequences of the mutation. Selective degradation of mutant proteins through Hsp90 inhibition has been documented many times for different genes relevant for tumour progression [22]. This de novo dependency links a previously robust trait to environmental fluctuation, rendering, in our case, eye development more susceptible to external perturbation. Partial penetrance of another *PAX6* homeodomain mutation (R242T) was also observed in the case of a child with unilateral iris coloboma, whose mutation carrier mother was completely unaffected [34]. Nonconservative missense mutations in *SUFU* (orthologous to the gene mutated in the zebrafish *dre* mutants) have been observed in independently isolated medulloblastomas [73] and are therefore associated with cancer. Geographical clusters of children with eye malformations, born to unaffected parents, and the increased incidence of some cancers in certain areas suggest that environmental factors may be implicated in these diseases [74,75]. Might such factors play their role by affecting the penetrance of underlying mutations or deleterious polymorphisms in susceptible individuals through diversion of Hsp90 function? While the modifiable eye mutants are caused by missense mutation, other Mendelian mutants that proved to be insensitive to mild Hsp90 reductions were due to stop codon mutations. We are currently investigating whether different types of mutations (truncation versus missense) at a single susceptible locus might have different susceptibility to Hsp90 buffering. Missense mutations within different protein domains (e.g., homeodomain versus paired domain in Pax6) of susceptible proteins may also confer different basal sensitivity to Hsp90 inhibition, as already demonstrated for some kinases [76,77]. Similarly, might loci initially resistant to Hsp90 fluctuation become susceptible in different genetic backgrounds?

The identification of Hsp90-buffered genes, mutation types, or alleles, and the structural basis of their sensitivity to limiting chaperone capacity have important clinical implications and will provide unique opportunities to investigate the regulatory mechanisms involved. Our findings open the way to the discovery of rational protective measures that could significantly reduce recurrence of severe developmental malformations.

## Materials and Methods

### D. rerio stocks.

Breeding fish were maintained at 28 °C in a circulating water system on a 14-h light/10-h dark cycle. Embryos were collected by natural spawning and were staged according to Kimmel [78]. The mutants *sunrise (sri), dreumes (dre), you too (yot),* and *sonic you (syu)* were isolated in a large an ENU mutagenesis screen [43] and were obtained from the Tuebingen Zebrafish Stockcenter [79], together with the WT strain Tuebingen. *AB, WIK, Albino, and Golden are commonly used WT strains and were originally obtained from the Zebrafish International Resource Center at the University of Oregon [80]. Albino and Golden strains have mutations affecting only melanophore pigmentation [81–82].

### Mating strategies.

Pair mating is between two identified individuals. Incross is mating between siblings. Outcross is mating between different mutation carriers or different strains. Mutant stocks were maintained by alternate breeding strategies between the parental Tuebingen strain and *AB, WIK, or Golden WT strains except for subsequent inbreedings. All the Mendelian mutants used in this study are recessive. *sri* mutants were found to be homozygous viable and fertile, so that the Hsp90 inhibition study could be carried out using genetically homogeneous homozygous mating pairs. Other Mendelian mutants were studied by mating proven heterozygotes, which gave rise to 25% affected offspring. For *yot,* molecular analysis was used to confirm genotype and segregation of AMN eye defects among F1 embryos. Inbreeding of AMN line was carried out for at least four generations by successive incrossing of identified pairs that produced AMN affected embryos with or without Hsp90 inhibition.

### Treatment of D. rerio embryos with Hsp90 inhibitors and heat shock.

Embryos collected from breeding pairs were incubated in a 0.025–0.1 mg/ml solution of protease (Type1, Sigma, http://www.sigmaaldrich.com) in system water, rinsed ten times, and partially dechorionated. Treatment was initiated at 30, 50, or 75% epiboly [78] and left overnight at 28 °C, followed by drug wash-out using five rinses. Larvae were examined between 24 and 120 hpf or up to 5 dpf, anaesthetised in a 0.1% solution of tricaine (Sigma), and immobilised in 4% methylcellulose (Sigma) where appropriate. To avoid variability between experiments, each spawning was split in two groups, and half was treated, while the other half was kept as its respective control (treated only with the relevant solvent). Radicicol (Sigma) was dissolved in 100% ethanol. GMP (gift from P. Csermely) and 17AAG were dissolved in 100% DMSO or methanol. Surprisingly, DMSO alone increased the penetrance of the AMN phenotype in embryos from Group 1 females in a dose-dependent manner (unpublished data). Increasing doses of methanol did not alter the number of AMN-affected embryos derived from these females. Neither AMN Group 2-derived embryos nor the unrelated *sri*-derived embryos have altered penetrance or increased severity when grown in the presence of DMSO or methanol compared to untreated siblings (Table 4). For Group 1-derived embryos 17AAG was therefore dissolved in methanol only. For all other mutants DMSO or methanol was used as solvents. Some of the controls were kept untreated (no solvent treatment and no dechorionation). Heat shock was carried out for 30 min at 37 °C in sealed petri dishes containing 30 ml system water floated on a water bath, followed by a return to 28 °C.

### Lithium treatment.

Lithium treatment was initiated at the same developmental stage as radicicol or 17AAG treatment. The embryos were incubated at 28 °C for 5–10 min in system water with 0.3 M lithium chloride, then washed extensively, and returned to 28 °C for further scoring at 24–72 hpf.

### *hsp90* morpholino microinjection.

The oligonucleotides were obtained from Gene-Tools (http://www.gene-tools.com). The sequences used were: *hsp90a,* 5′-TCTTTGTTGAATTATTCGCTGTATT-3′; *hsp90b,* 5′-TCGTTGATTTTTGATGTTTTAATCG-3′; Fluorescein STD control morpholino*,* 5′-CCTCTTACCTCAGTTACAATTTATA-3′.

The morpholinos were dissolved in water at a final concentration of 42 ng/nl. Working dilutions were made with 1% phenol red (*hsp90a* and *hsp90b* or STD control*).* Approximately 1.5 nl were injected into the cytoplasm of two to four cell stage embryos.

### Transfections and immunohistochemistry.

Mammalian expression vectors pCMV-ZfPax6b and pCMV-ZfPax6b*sri*, respectively containing the WT or mutant L244P version of the full-length Pax6b cDNA (gift of Dirk Kleinjan), were used in cotransfections with the GFP reporter pEGFP-C1 (Invitrogen, http://www.invitrogen.com). *Hela* cells were maintained in Dulbecco's modified Eagle's medium supplemented with 10% calf serum (Gibco, http://www.invitrogen.com). We seeded 10^5^ cells onto 22 × 22-mm coverglass 24 h prior to transfection. Next 200 ng of Pax6b WT or sri mutant vectors were cotransfected with 50 ng of GFP reporter using Lipofectamine2000 (Invitrogen) as indicated by manufacturer. Lipofectamine complex was replaced 4 h posttransfection with fresh DMEM media containing DMSO as solvent control, or increasing doses of 17AAG. Next day, immunohistochemistry was performed using standard methods. Mouse monoclonal anti-Pax6 antibodies [83] and rabbit polyclonal anti-GFP antibody (Santa Cruz, http://www.scbt.com) were used with fluorescently labelled second antibodies (Molecular Probes, http://probes.invitrogen.com). Images were captured and compared using IPLab. Pax6 (red) to GFP (green) ratios were calculated by manually counting the number of red cells that appeared in the same field as 100 green cells, at each condition. The ratio observed in Pax6b-WT and Pax6b-sri DMSO controls were set as 100%. The effect of Hsp90 inhibition on Pax6b proteins was calculated as the relative percentage to each of these respective controls.

### Western blotting.

Lysates were prepared from 20–50 larvae according to Westerfield [84]. Bradford Protein Assay (Biorad, http://www.bio-rad.com) was used to quantify protein levels for equal loading and confirmed by Coomassie blue staining of a duplicate gel. Western blot analysis was carried out using standard techniques. Specific analysis for the induction of a generalised stress response was carried out using Hsp70 antibody from Stressgen SPA-812 (http://www.nventacorp.com). α-tubulin monoclonal antibody TAT1 [85] was used as loading control. RAF1 levels were detected with Abcam ab19927 (http://www.abcam.com), a rabbit polyclonal antibody. ECL detection with relevant secondary antibody (Amersham, http://www.amersham.com) binding was carried out according to the manufacturer's instructions.

### Scoring of D. rerio mutant phenotypes.

Quantitative measurements of *sri* and *dre* were carried out using IPLab software (http://www.scanalytics.com) on digital photographs. In *sri* and *dre* embryos eye shape (eccentricity) was calculated as √[(major axis)^2^ − (minor axis)^2^]/(major axis) for the region of interest, where zero is a circle and 1 an extreme ellipse. The axes and area were calculated automatically after drawing around the outer edge of the retina or lens on the digital images. The presence of coloboma was noted. Individual values and distributions are displayed in the scatter plots (Figure 3). Mean values and statistical results are represented in Table 3.

Cryptic developmental eye defects were revealed, initially following Hsp90 inhibition with radicicol, among the offspring of *yot* (+/−) crossed to WT fish. Eye phenotypes were assessed in treated and control embryos at 48–120 hpf. Carrier parents were identified based on the repeated production of offspring with AMN-like eye phenotypes after radicicol treatment. F1 fish are the unaffected siblings from pair matings that produced significant numbers of embryos with AMN phenotypes. Some of these sibling offspring were individually pair mated, and each spawning divided into treated and control groups for subsequent phenotypic assessments. From F1 onwards, embryos were treated with 17AAG dissolved in either DMSO or methanol as described above. The same analysis was also carried out for nontransmitters derived from the *yot* population, from *sri* and *dre,* as well as from the genetically distinct *WT* strains, *WIK, Golden,* and *Tuebingen* (Table 2; unpublished data)*.*

Abnormal eye structure with reduced eye size is classified as microphthalmia (Figure 4B) and complete absence of eye as anophthalmia (Figure 4A and 4E). Nanophthalmia is defined as normal eye structure but reduced size (Figure 4C). Each of these mutant phenotypes was classified as unilateral (one eye affected and one eye WT) or bilateral (both eyes equally or unequally affected). For the assessment of developmental stability, cyclopia is considered as a bilateral defect.

Laterality of cardiac heart looping was assessed on ventral view of partially anesthetized 48–72 hpf live embryos under an inverted microscope. Movies were taken of some examples of normal and aberrant looping (see Supporting Information).

### *yot* genotyping.

Genomic DNA was extracted from whole embryos or adult tails as described [49]. The *gli2* gene was amplified by PCR with specific primers and the product analysed by direct sequencing. The oligonucleotides: 5′-GAGCCTTAAAACTAGAATGGCCA-3′ and 5′-CCATCAGTGGCCATATTTTCC-3′ were used as forward and reverse primers, respectively.

### Statistics.

 To assess the statistical significance of the phenotypic distribution of retina to lens ratio and shape, a two-sample *t*-test, under the assumption of unequal variances, was used to compare the means of the groups (Tables 2–4). Because of the variable penetrance of the AMN trait in different egg lays and pairs, the effect of Hsp90 inhibition on this phenotype was calculated by combining the data from several crosses (Tables 5 and 6) as a weighted mean of the difference between the proportions of affected embryos in the treated and sibling control groups over each cross. The square of this statistic has a Chi^2^ distribution with one degree of freedom. For lithium susceptibility studies, a two-tailed unpaired Fisher's exact test was used to assess whether differences between solvent control and treated siblings were significant (Figure 7). Frequency of heart-loop inversion was analysed by Chi^2^ test with one degree of freedom (Figure 8). For developmental stability of quantitative eye traits in WT strains, a two-tailed unpaired Fisher's exact test was used to assess whether differences in left and right measurements between control and treated siblings were significant. For *sri-*derived embryos an ANOVA analysis of the logarithm of the absolute difference of the left and right measurements normalized to the mean trait value within each individual was corrected for batch and presence of coloboma and used to asses difference between control and treated siblings ( | log_10_(L − R)/(L + R)/2) | ). Only those embryos with mild coloboma where the lens could be accurately measured were counted in the analysis.

## Supporting Information

Figure S1 Frequencies of Common Developmental Defects Elicited in WT Strains by Decreasing Hsp90 Function at 30% or 50% Epiboly 17AAG treatment (3.3 μM) initiated at: 30% epiboly (violet) or 50% epiboly (green).
(39 KB PPT)

Figure S2 Abnormal Phenotypes in Morpholino Injected Embryos (A) Absence of looping of the heart tube in Hsp90 morpholino-injected embryos.
(B) Severe oedema in Hsp90 morpholino-injected embryos.
(27 MB TIF)

Video S1 In Some Severe Cases Drug-Induced Oedema Leads to Abnormal Heart Morphology (1.2 MB MOV)

Video S2 Normal Heart Looping At around 22–30 hpf, the heart tube is gradually bent to form an s-shaped loop that positions the ventricle to the right of the atrium; this is in turn the first morphological indication of the left-right asymmetry in the zebrafish embryo.
(591 KB MOV)

Video S3 Initiating 17AAG Treatment at 30% Epiboly Leads to High Frequency of Heterotaxia Heart chambers develop normally but the direction of the loop is inverted, positioning the ventricle to the left of the atrium. Most of these fish were viable and fertile (unpublished data).
(3.2 MB MOV)

Video S4 Radicicol-Induced Heterotaxia (621 KB MOV)

Video S5 Absence of Looping of the Heart Tube in Radicicol-Treated Embryos (I) (1.2 MB MOV)

Video S6 Absence of Looping of the Heart Tube in Radicicol-Treated Embryos (II) (1.7 KB MOV)

### Accession Numbers

The RefSeq (National Center for Biotechnology Information [NCBI]) (http://www.ncbi.nlm.nih.gov/RefSeq) accession numbers for the genes discussed in this paper are for human: *Hsp90AA1* (NM_001017963, NP_001017963.1/.2); *Hsp90AB1* (NM_007355.2, NP_031381.2); *Hsp90B1* (NM_198210, NP_937853); *TRAP1* (NM_016292.1, NP_057376.1); and for zebrafish: *hsp90a* (NM_131328.1, NP_571403.1); *hsp90ab1* (NM_131310.1, NP_571385.1); *pax6b (*NM_13164.1, NP_571716.1); *sufu* (XM_689570.1, XP_694662.1); *gli2 (gli2a)* (NM_130967.1, NP_571042.1); and *shh (shha)* (NM_131063.1, NP_571138.1).

## Abbreviations

17AAG: 17-allylamino-17-demethoxygeldanamycin
AMN: anophthalmia, microphthalmia, and nanophthalmia
dpf: days postfertilization
GA: geldanamycin
GMP: geldampicin
hpf: hours postfertilization
Hsp70: heat shock protein 70
Hsp90: heat shock protein 90
Hsp90 a+b: Hsp90 alpha and beta antisense morpholinos
WT: wild type
