# Supplementary material for: Hsp90 Selectively Modulates Phenotype in Vertebrate Development
Source: PLoS Genet. 2007 Mar 30;3(3):e43. doi: 10.1371/journal.pgen.0030043 (PMC1839141; doi:10.1371/journal.pgen.0030043)
Supplement: Figure S1 — 17AAG treatment (3.3 μM) initiated at: 30% epiboly (violet) or 50% epiboly (green). (39 KB PPT) [file pgen.0030043.sg001.ppt]

## Slide 1
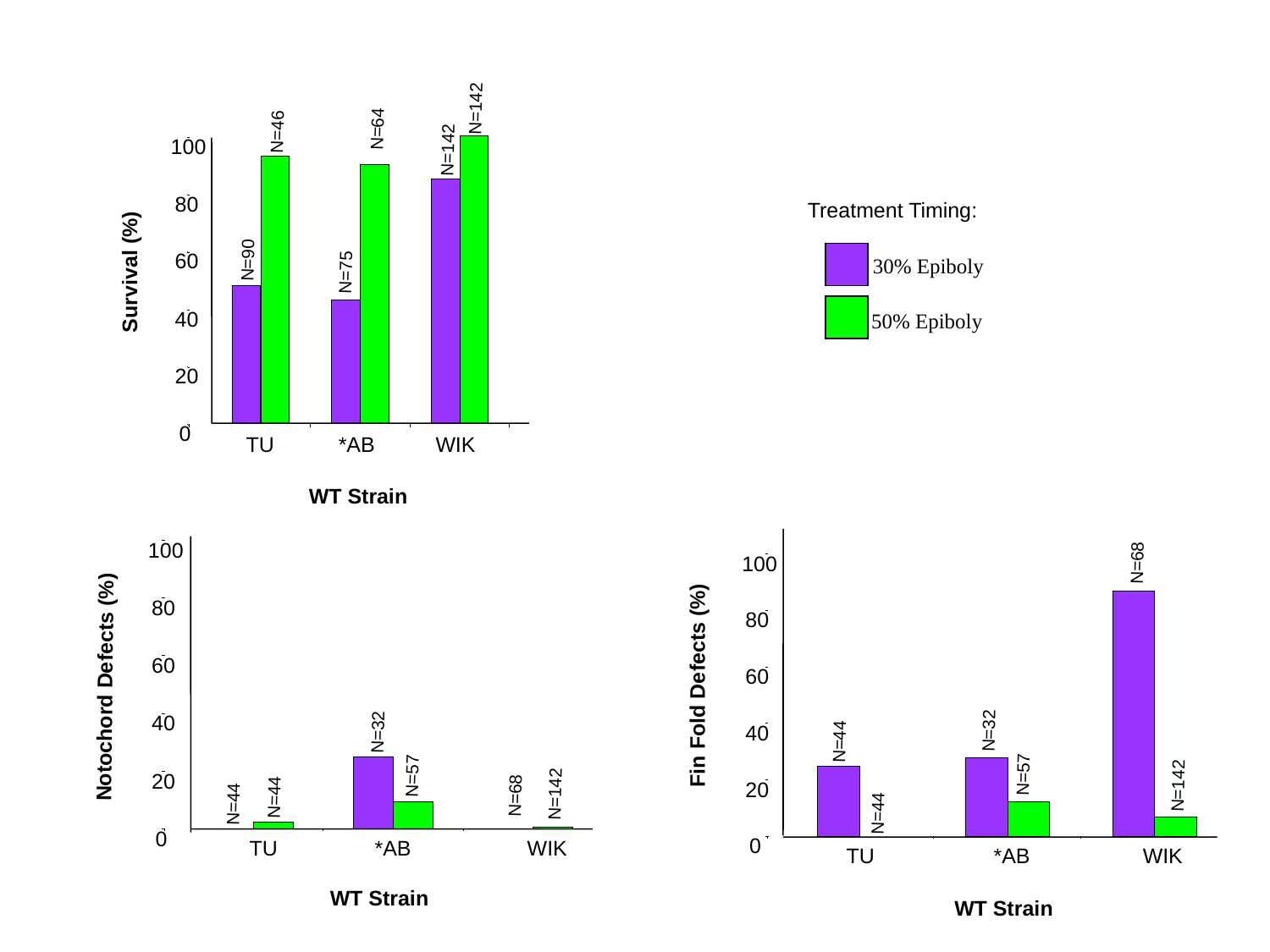

N=142
N=64
N=46
N=142
100
Treatment Timing:
80
N=90
30% Epiboly
60
N=75
Survival (%)
50% Epiboly
40
20
0
TU
*AB
WIK
WT Strain
100
N=68
100
80
80
60
60
Fin Fold Defects (%)
Notochord Defects (%)
40
N=32
N=32
40
N=44
N=57
N=57
20
N=142
20
N=142
N=68
N=44
N=44
N=44
0
0
TU
*AB
WIK
TU
*AB
WIK
WT Strain
WT Strain
