# Supplementary figures and images for: Hsp90 Selectively Modulates Phenotype in Vertebrate Development
Source: PLoS Genet. 2007 Mar 30;3(3):e43. doi: 10.1371/journal.pgen.0030043 (PMC1839141; doi:10.1371/journal.pgen.0030043)

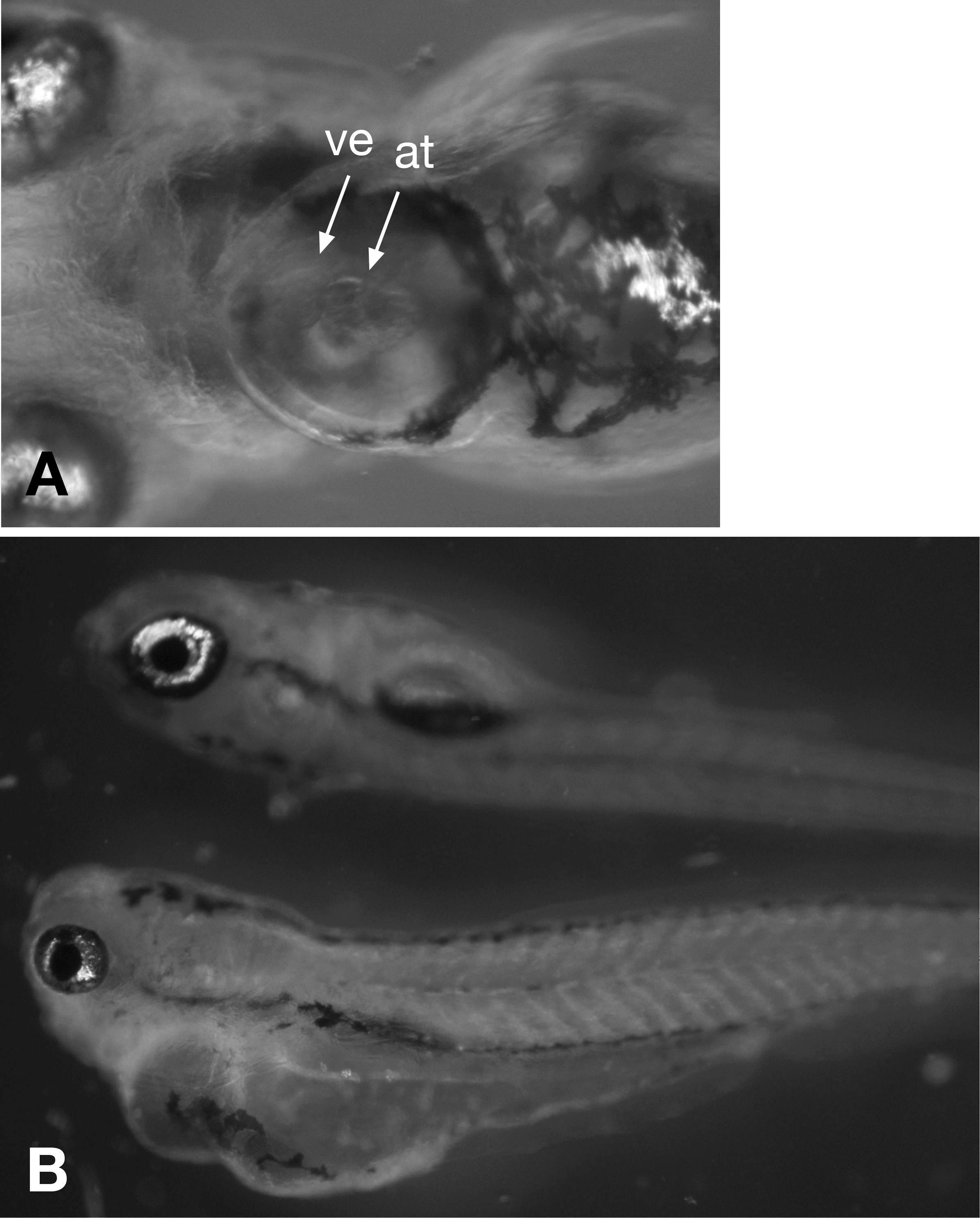

Supplement: Figure S2 — (A) Absence of looping of the heart tube in Hsp90 morpholino-injected embryos. (B) Severe oedema in Hsp90 morpholino-injected embryos. (27 MB TIF) [file pgen.0030043.sg002.tif]
